# Redistribution of Doctors and Decentralization of Clinics Improved Utilization of Services, Demand, and Capacity of Hamad Medical Corporation’s Staff Clinic

**DOI:** 10.7759/cureus.25883

**Published:** 2022-06-12

**Authors:** Elmukhtar Habas, Anas M Al Halabi, Maliha S Saleem, Hafedh Ghazouani, Ahmed A Hommos, Abdelsalam M Borham, Abdul-Badi Abou-Samra

**Affiliations:** 1 General Internal Medicine, Hamad General Hospital, Doha, QAT; 2 Quality and Patient Safety, Hamad Medical Corporation, Doha, QAT; 3 Internal Medicine, Hamad Medical Corporation, Doha, QAT; 4 Medicine, Hamad Medical Hospital, Doha, QAT

**Keywords:** waiting time, physician redistribution, patient flow, staff clinic, simulation

## Abstract

Background: The Staff Medical Clinic (SMC) of the Hamad Medical Corporation (HMC) serves the staff members who require healthcare services, but in a crowded environment, the SMC can only meet 75% of that demand. Overcrowding reduces productivity and service quality and increases waiting time. Furthermore, overcrowding in healthcare facilities decreases the experience and satisfaction of patients and healthcare providers.

Aim: The main objective of this study was to use simulation modeling to evaluate interventions that could improve SMC waiting time and efficiency.

Method: Eighteen months of data on SMC patient flow, staffing, and clinical sessions were collected (January 2018 to June 2019). The patient's journey through the SMC was modeled as a series of processes with assigned durations defined mathematically using the appropriate probability distribution. A simulation flow model was developed considering the locations of the staff and nearby main hospital facilities. An intervention was proposed and evaluated through a simulation. The intervention involved redistributing 25% of the SMC staff into three main satellite clinics located at the facilities where most of the SMC patients came.

Results: The proposed intervention decreased crowding by 37%, reduced staffing requirements by 28%, and increased the number of patient slots by 22%, resulting in a net increase in the number of patients served by an average of 1250 monthly, without the need for hiring new additional staffing.

Conclusion: Redistribution of the available medical staff to three new satellite clinics reduces workload pressure at all sites and increases clinic capacity without additional costs.

## Introduction

Overcrowding occurs when the demand for a service exceeds the available capacity, resulting in long waiting times. Overcrowding and long waiting times increase the risk of hospital-related problems, such as infection transmission; reduce patient satisfaction and experience [1]; and adversely influence patient outcomes [2-5], working atmosphere, and staff effectiveness [1].

The complexity of a healthcare system can be simulated in a realistic setting [2-5]. Simulations allow the discovery of the system defects and an understanding of their overall impacts. The simulation also enables the testing of suggested interventions and predicting their outcomes with high accuracy. Simulation scenarios may be used to analyze groups of behaviors and the impact of implementing new protocols. Outcomes from these simulations help healthcare professionals and other decision-makers address real-world healthcare challenges [6]. Errors during simulation provide opportunities to learn and achieve the competencies, reducing harmful incidents and adverse outcomes before applying the changes in the system [6].

Simulation is an established method that uses a what-if scenario analysis tool in operational research. Discrete event simulation (DES) is a technique used to model the evolution of the status of an entire system at specific time points. A model is a “simplified structure of reality,” representing the characteristics or relationships assumed to be significant for a given problem. This model's relevant elements and mechanisms are formalized using parameters and variables. DES is an approach with good motivation: improvement of processes and service delivery in the healthcare system. The so-called agents (conditions and action rules) characterize the behavior and determine the system's evolution from a given initial sociospatial configuration [7]. The parameterizing model assigns a numerical value to formalize the model by calculating the variables, making the simulation more realistic. Parameterization is the process of setting important values as a model's structure for calibrating and getting a good simulation's accuracy. It consists of parameters indicating the specific attributes of a system to explore the best fit. These are based on accurate data by estimating them using one of the relevant statistical models to accommodate more realistic scenarios. Parameterization includes location parameters, individual, and resource allocations. The DES model was used when it had 50 replications or more, which was sufficient to distinguish the scenario's meaning and compare it with the actual measurement mean for a reference period. Generally, forecasting tools and techniques are used to evaluate scenarios based on analysis [8].

Hamad Medical Corporation (HMC) is Qatar's main public health service provider. HMC manages nine main hospitals, three community hospitals, the National Ambulance Service, and home and residential care. It established Qatar's first academic health system, combining advanced research, education, and clinical care. HMC's research environment has made Qatar one of the four best Arabic countries in research paper publication [9].

The Staff Medical Clinic (SMC) provides a wide range of medical services for the medical needs of all staff members who have a contract with the HMC. Although the main SMC is usually overcrowded, it meets approximately 75% of the demand. This study aimed to provide a logical approach to identify operational interventions that could reduce overcrowding and workload pressure at the Medical Staff Center (MSC) and meet the demands without extra cost.

## Materials and methods

**Figure 1:**
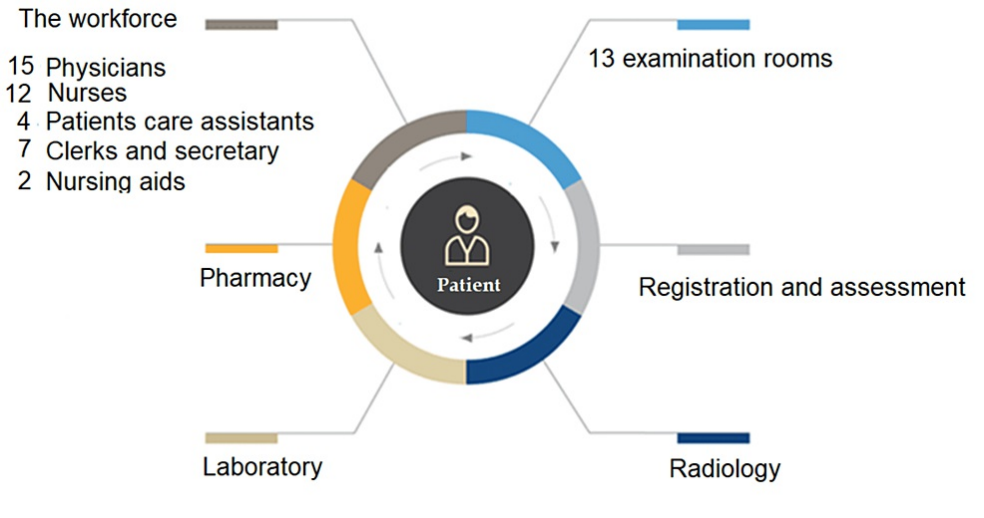
Resource definition of the Staff Medical Clinic (SMC) and patient journey within SMC

The main SMC serves the everyday clinical needs of the medical and paramedical staff and other support staff of the HMC. The main SMC is staffed by 15 doctors from different specialties and has 13 outpatient clinic rooms. Ten rooms were dedicated for patient examination, two for assessment, and one for vaccination. The main SMC has supportive accessories such as laboratories, radiology, and pharmacies to deliver patient-centered care (Figure 1).

Data for each subprocess of the consultation time (operational time [OT]) were collected over 18 months (January 1, 2018, to June 30, 2019) by direct observations, interviews, and electronic medical records (Cerner). The collected data included arrival time, registration, clinic start, consultation period, discharge, and nonattendance rate. Additionally, physicians' availability, number of doctors' offices, and environmental factors (such as seats and waiting space) that can affect the SMC productivity were assessed. The data were collected based on health service encounters and not on the patient's name. The collected data depended on the number of visits, regardless of the visitor. For example, a staff member visited the main SMC more than once during the study period, and each visit was counted (i.e., a new encounter). The real status of the SMC is shown in Table 1.

**Table 1:** Real status of the Staff Medical Clinic ^a^Mean (minimum-maximum). ^b^Mean ± Standard deviation.

Parameters	Values
Number of sessions per day^a^	15 (11-18)^a^
Number of patients per day^a^	220 (167-256)^a^
Number of physicians (fixed number)	15
Average monthly demand (monthly)^a^	6337 (5781-7084)^a^
Average production (patients seen monthly)^a^	4650 (3911-5172)^a^
The highest number of patient visits per month based on full capacity	4592
Deficit (demand versus production) on a percentage	-27%
Surplus (production versus capacity) on a percentage	+5%
Mean time (minutes) spent for service (time from entering to leaving the facility)^b^	29.1 ± 3.4^b^
Mean time (minutes) spent by a patient at the doctor's office^b^	14.7 ± 1.2^b^

## Results

**Figure 2:**
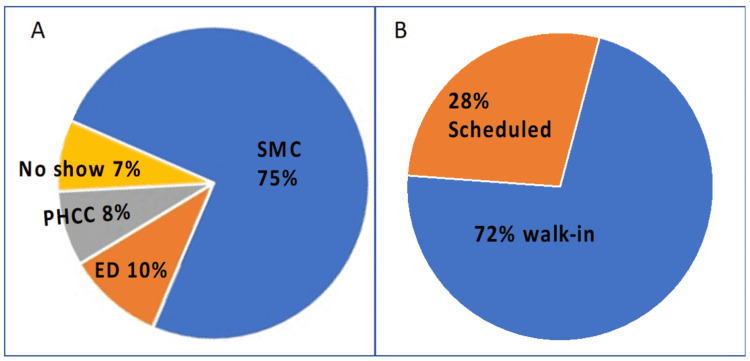
Staff clinic volumes and access by visits during the study period (A) shows that Staff Medical Clinic (SMC) served 75% of the demand during the observation period; the remaining served at the emergency department (ED), the Primary Healthcare Corporation Center (PHCC), or did not show up for their appointment. (B) shows that most patients served at the SMC were walk-ins, and only 28% were prescheduled.

The total number of encounters was 107,821 during the 18-month study interval (5990 monthly), but only 80,710 patients (75%) received medical service. Among the remaining, 10% received services at the emergency departments (EDs), 8% at the primary healthcare center (PHCC), and 7% had an appointment but did not attend (Figure 2, Panel A). Most patients were walk-in same day service (72%), and only 28% of them had a prescheduled appointment (Figure 2, Panel B).

**Figure 3:**
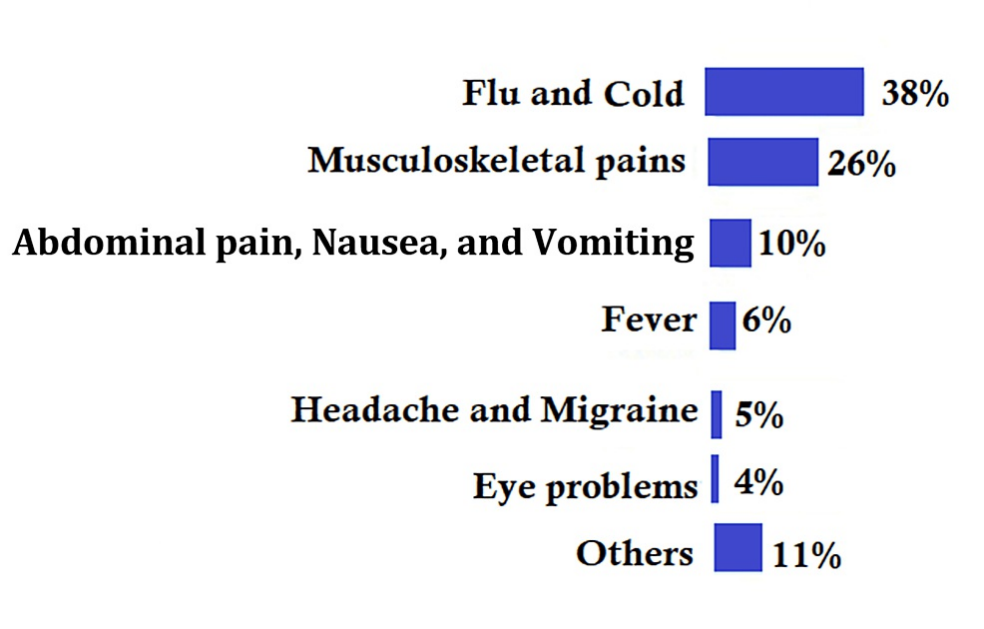
Principal causes of visits to the staff clinic

The principal causes of visiting the SMC included upper respiratory tract infections (38%), musculoskeletal pains (26%), abdominal pain, nausea, vomiting (10%), and others (Figure 3).

**Figure 4:**
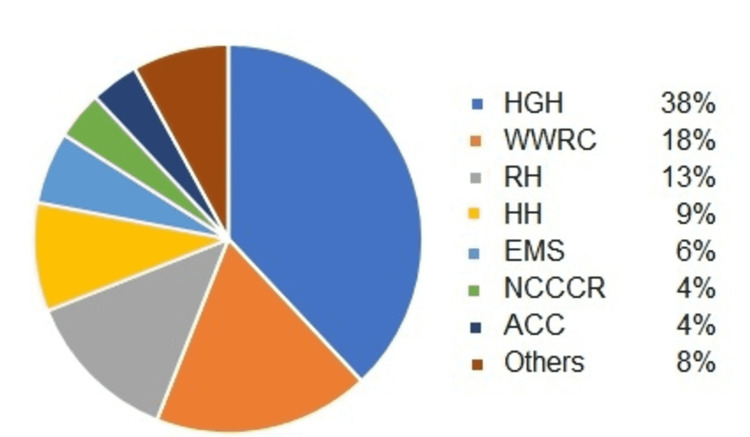
Sources of patients HGH: Hamad General Hospital; WWRC: Women Wellness and Research Center; RH: Rumaillah Hospital; HH: Heart Hospital; EMS: Emergency Medical Services; NCCCR: National Center for Center Care and Research; ACC: Ambulatory Care Center.

The majority of patients attending the SMC were staff from HGC (38%), the Woman Wellness and Research Center (18%), and Rumaillah Hospital (13%) (Figure 4).

**Figure 5:**
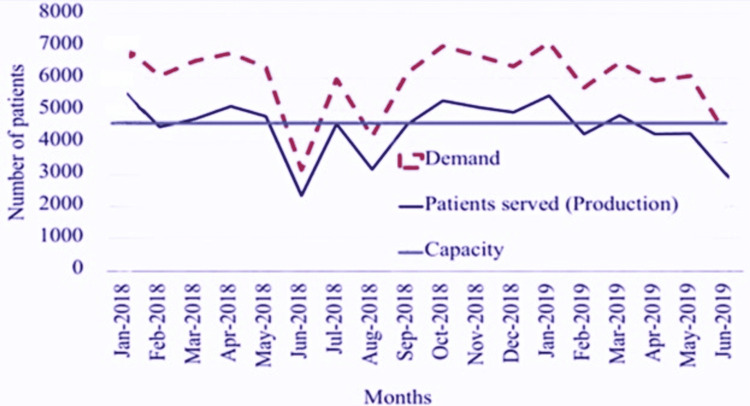
Monthly capacity and demand over the 18-month observation period

Of the 80,710 patients served at SMC, only 89% were served with the existing clinical capacity used at 100%, whereas 11% were served over the maximum available capacity (extra working hours paid to existing staff); the demand exceeded capacity during all the observation period (Figure 5).

**Figure 6:**
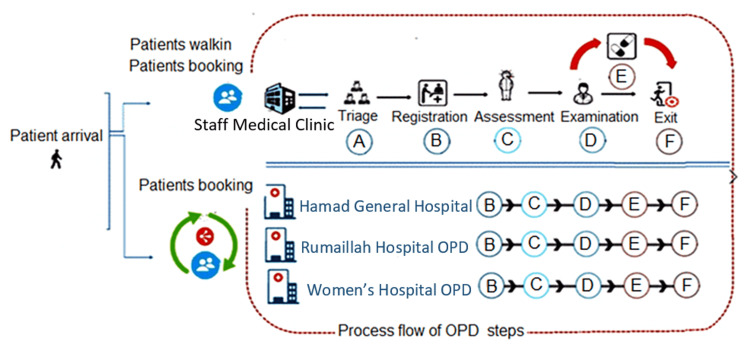
Context diagram of the patient flow in the simulation model The graph shows the system or process that was designed for walk-in patients (i.e., without appointment) and patients booking (i.e., with appointment). OPD: Outpatient department.

As most of the SMC patients derive from the three largest HMC facilities (Figure 4), a scenario was conceived to open a satellite SMC examination room at each of the three main nearby facilities to support the main SMC using available SMC staff (Figure 6).

To implement the suggested scenario, 25% of SMC staff were redistributed to the three facilities. The simulation showed improved consultation time and increased capacity by 37%, without the need for additional staff or extra working hours and without significantly affecting the resource allocation (material, staff, or building in the main SMC). The clinic space, nurses, and the registration staff in the satellite clinics were provided by the staff in the three hospitals, while the main SMC provides only the physicians. Table 2 represents the simulation scenario outcome.

**Table 2:** Simulation scenario results The data are averages of numbers or percentages, as appropriate.

Scenario	Production	Resource utilization		Capacity utilization (%)
	Patients served monthly (N)	Doctors (%)	Exam rooms (N)	
Baseline	4152	63%	10	100%
Adding three satellite clinics	5082	81%	13	72%
Improvement %	22%	18%	30%	28%

**Figure 7:**
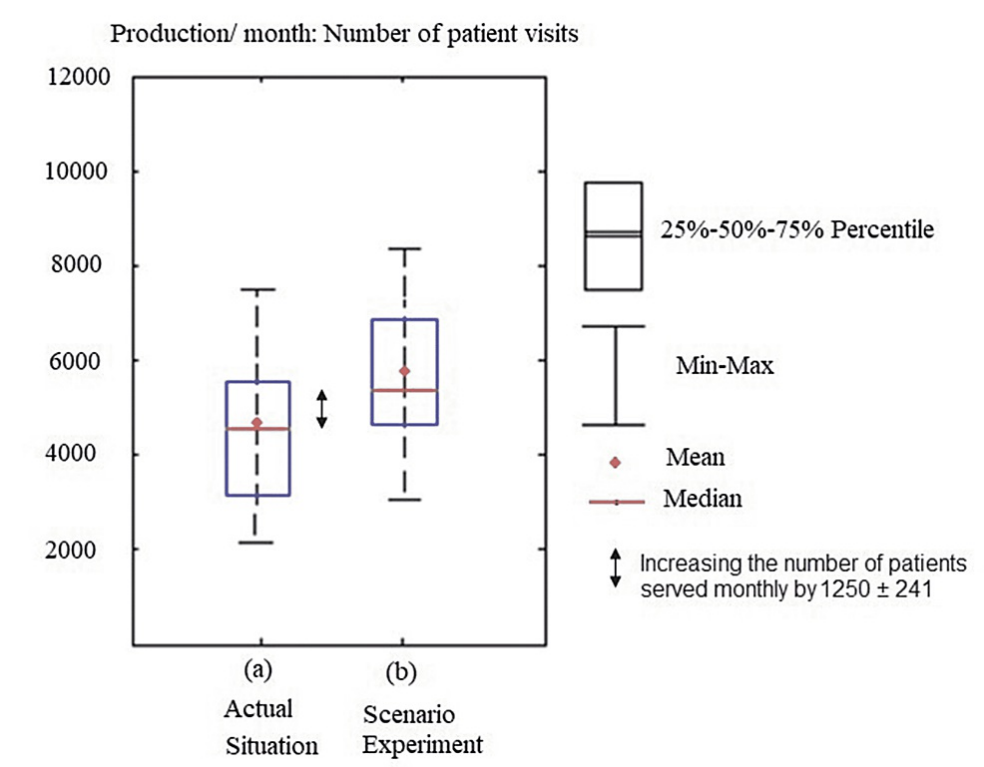
Scenario: graphical results of the experiment The graph shows average monthly staff visits in the "Actual scenarios" (a) and the "Scenario experiments" (b). The boxes show the interquartile ranges, while the horizontal lines represent medians. The vertical lines depict the maximum and minimum values. They were then compared to an average monthly measure. The figure shows that there is an increase in the number of patients served per month by 1250 ± 241.

According to the DES simulation, the results showed very little change in the time spent on the patient journey (0.3%) but a highly valuable increase in production (22%). These results support that a simulation model is a valuable tool for assessing interventions even before applying them [9]. The total demand and production trajectories were unstable in the actual data, exceeding the maximum capacity limits that fit the range of monthly demand versus capacity from 975 to 1745. Following the simulation implementation, the time service was significantly improved, making the demand and production trajectories fall under the ultimate capacity line. The model demonstrated a significant increase in staff utilization after staff clinic decentralization by 37% and increased the number of patients served monthly by 1250 ± 241 (Figure 7).

## Discussion

Understanding the pattern of SMC in the service scope context and challenges in service delivery using the simulation scenario enables the design of an appropriate service depending on the needs of the staff working in the HMC. Modeling helps identify areas that require improvement and test alternative solutions and highlights the benefits of modeling operational changes before implementation, particularly in resource-limited situations [7].

The global capacity utilization benchmark is the maximum production level of goods and services that a given system can achieve over a specified period. The percentage of staff members examined with the current system was >5%, with maximum utilization of the capacity of available resources. Despite the total usage of the available resources, 25% of the attendees were not well served within the main SMC, leading them to attend other facilities and attend nonmedical services, such as approving sick leave. This implies that almost 30% of the extra load is imposed on the working staff. This may cause inconvenience to staff, poor satisfaction with the SMC service, and impaired workplace productivity. The most common reasons that cause patients to shift to other facilities to have the needed services are a lack of accessibility to the centralized staff of medical services, overcrowding, limited resources, limited sitting places, parking spaces, and the presence of all services on site. These factors may lead to the staff members not attending the SMC. Therefore, staff members participate in other facilities to avoid these factors and increase turnaround time.

Optimizing the SMC workforce, improving follow-up scheduling, reducing pressure on the existing service, and improving the quality of the service delivered will improve SMC's productivity and staff members' satisfaction. The quantitative data results show a reduction of approximately 10%, implying that improving patient flow by understanding needs can address the challenges of strengthening service utilization.

The study's statistical data showed that the number of patients seen by a physician per session was reduced by physician sessions (15 vs 13), as demonstrated by the simulation, allowing adequate time for proper care and reducing the workload of SMC staff. Furthermore, the intervention increased physician efficiency and utilization of the service by reducing the wastage of appointment slots [8]. Moreover, the establishment of satellite clinics has allowed the provision of complementary services as per the scope of the SMC, which can aid in providing efficient services to patients on time, improve patient flow in the main SMC, and improve staff HMC satisfaction. Additionally, this study explores an ideal scenario simulation that can be used to address challenges to the efficient restructuring of the outpatient department setting, providing the best possible services [10-12].

Limitations

Although the study was conducted in SMC in Hamad Medical City - Qatar, applying this intervention to other places may need some changes; however, redistribution and perfect use of the available resources will improve the service and not significantly affect the cost. In addition, all simulation processes can only be proven when applied in the real world.

## Conclusions

Patient overcrowding, extreme resource limitations, and healthcare workforce shortages risk patient safety. Predicting the risk factors and simulating them may help make plans to overcome health crises. Our simulation results show that physician redistribution and a resource-sharing environment based on the decentralization of services by satellite staff clinics reduce the main SMC overcrowding and decrease workload without significant extra resources. This intervention can reduce the potential for adverse outcomes and improve employee experience and satisfaction.
